# UK obstetric sonographers’ experiences of the COVID-19 pandemic: Burnout, role satisfaction and impact on clinical practice

**DOI:** 10.1177/1742271X221091716

**Published:** 2022-05-14

**Authors:** Emily Skelton, Gill Harrison, Mary Rutherford, Susan Ayers, Christina Malamateniou

**Affiliations:** 1 City, University of London, London, UK; 2 King’s College London, London, UK; 3 The Society and College of Radiographers, London, UK

**Keywords:** Burnout, COVID-19, obstetrics, job satisfaction, sonographer, well-being

## Abstract

**Introduction:**

The COVID-19 pandemic placed additional demands and stressors on UK obstetric sonographers, who were required to balance parent safety and service quality, alongside staff safety. Increased pressure can negatively impact a healthcare worker’s well-being and the provision of person-centred care. The aim of this study was to explore obstetric sonographers’ experiences of performing pregnancy ultrasound scans during the pandemic and to assess the impact on burnout, role satisfaction and clinical practice.

**Methods:**

An online, anonymous cross-sectional survey was created to capture sonographers’ experience alongside using the Oldenburg Burnout Inventory to evaluate burnout and Clinical Outcomes in Routine Evaluation 10 (CORE-10) to measure psychological distress.

**Results:**

Responses were received from 138 sonographers. Of those completing the Oldenburg Burnout Inventory (*n* = 89), 92.1% and 91.0% met the burnout thresholds for exhaustion and disengagement, respectively. Sonographers with a higher burnout score also perceived that COVID-19 had a greater, negative impact on their practice (*p* < 0.05). The mean CORE-10 score of 14.39 (standard deviation = 7.99) suggests mild psychological distress among respondents. A significant decrease in role satisfaction was reported from before to during the pandemic (*p* < 0.001), which was associated with higher scores for burnout and psychological distress (*p* < 0.001). Change in role satisfaction was correlated with sonographers’ perception of safety while scanning during the pandemic (*R*^2^ = 0.148, *p* < 0.001). Sixty-five sonographers (73.9%) reported they were considering leaving the profession, changing their area of practice or working hours within the next 5 years.

**Conclusion:**

Job and context-specific interventions are required to mitigate burnout and its consequences on the workforce and service provision beyond the pandemic.

## Introduction

Occupational burnout syndrome is a psychological phenomenon defined as a ‘prolonged response to chronic emotional and interpersonal stressors on the job’.^1^ The development of burnout in obstetric sonographers can be explained using the job demands-resources model, which identifies two processes leading to the burnout domains of exhaustion and disengagement. In the ‘job demands’ process, exhaustion is a consequence of sustained physical and/or psychological work pressures (e.g. heavy workload, interpersonal interactions, sub-optimal work environment).^2^ Demands specific to obstetric sonographers, which also contribute to the exhaustion domain, include unexpected news delivery in cases of foetal anomaly or miscarriage,^3^ maintaining concentration while experiencing distractors in the scan room,^4^ as well as the physical exertion of scanning a population with increasing body habitus.^5^ Meeting these demands can be made more challenging by a lack of ‘job resources’, including support from supervisors, and opportunities for personal growth, which can lead to disengagement from work.^6^

High and rising levels of burnout in healthcare practitioners, including sonographers, have been previously acknowledged.^7,8^ Additional stressors of the COVID-19 pandemic (e.g. lack of personal protective equipment (PPE), fear of contracting or transmitting the virus, or working under rapidly changing guidelines) may also have a negative psychological impact on healthcare workers;^9^ thus, there is potential for the proportion of sonographers meeting the threshold for burnout post-pandemic to be even higher than previously reported. The consequences of burnout on healthcare professionals are well-known, with established associations between the syndrome, mental health, job performance and patient care.^10^ During obstetric ultrasound scans, the parent–sonographer partnership is integral to support the delivery of parent-centred care; however, there is limited research into burnout in medical imaging professionals,^8^ and even less regarding the specific impact of sonographer burnout on parental experiences of foetal ultrasound.^11^

An additional challenge faced by obstetric sonographers during the pandemic was that many clinical departments temporarily restricted the attendance of partners and support persons at scans in an attempt to minimise virus transmission.^12^ In addition to the clinical requirements of the examination, foetal ultrasound scans are often regarded as a milestone event in pregnancy, which provide expectant parents with an opportunity to see their unborn baby. While most parents were understanding of these measures, the profession received critical media attention from expectant parents, other health care staff and parent advocacy groups,^13^ which may have contributed to further stress in sonographers.

The aim of this study was to explore sonographers’ experiences of performing obstetric ultrasound examinations in the UK during the COVID-19 pandemic to further understand the impact of the pandemic on sonographer burnout and psychological well-being, and consider the implications on the sonographic workforce.

## Methods

A UK-wide, cross-sectional open survey design was used to collect data from an anonymous, online questionnaire, created using the secure Qualtrics XM^TM^ survey platform (www.qualtrics.com). The Checklist for Reporting Results of Internet E-Surveys (CHERRIES) was used to guide the reporting of the survey methods and results.^14^ This 30-item checklist helps to standardise the reporting of web-based surveys to enable readers to identify potential bias in the methods and establish their own conclusions about the validity of the findings. The questionnaire was divided into four sections: Part 1 captured sonographers’ experiences of obstetric scanning during the COVID-19 pandemic, parts 2 and 3 used the validated Oldenburg Burnout Inventory (OLBI) and Clinical Outcomes in Routine Evaluation 10 (CORE-10) tools to evaluate and measure sonographer burnout and psychological distress, respectively, and part 4 recorded basic demographic information (e.g. age, geographical location and employment status). Where appropriate, open-ended questions were used (e.g. for participants to provide additional detail if they wished to). These free-text responses will be qualitatively analysed and included in a separate publication as part of the larger doctoral research project (www.blogs.city.ac.uk/afi-study). The questionnaire was piloted for usability with members of the Society of Radiographers Ultrasound Advisory Group. Their recommendations for minor changes to the wording and display of some questions were incorporated into the final version, prior to launch, for improved accessibility. Participants were prompted (but not forced) to answer all questions and were given the option to review and change answers using navigation buttons within the survey. As the survey contained a mixture of response types (e.g. single click vs free text), no restrictions were placed on the time allotted for completion. To ensure anonymity, no directly identifying participant information was collected. The survey was designed so that participants were prevented from attempting to complete it more than once.

The questionnaire was live for 8 weeks between 9 March and 6 May 2021. The recruitment strategy used snowball sampling via social media channels (Twitter, Facebook, LinkedIn) and word-of-mouth through professional networks to circulate a weblink to the questionnaire. Participants were required to meet all of the following inclusion criteria to be eligible to take part: (1) a qualified sonographer/ultrasound practitioner who has performed obstetric ultrasound scans in the UK since March 2020 (e.g. during the COVID-19 pandemic), (2) aged ⩾21 years and (3) informed consent form completed. No incentives were offered to participants. The data collection period coincided with the UK’s third national lockdown which began on 6 January 2021.^15^

### Oldenburg Burnout Inventory

The OLBI comprises 16 items covering two dimensions: exhaustion (OLBI-E) and disengagement (OLBI-D) from work, which reflect the physical and cognitive aspects of occupational burnout. The highest burnout response to each item scores 4 points, and the lowest scores 1 point. The total burnout score was recorded, and the average scores for each dimension were calculated and compared against a threshold of ⩾2.25 for exhaustion and ⩾2.10 for disengagement, which have been previously used to determine burnout in other studies.^16–18^

### CORE-10

The CORE-10 is a short, generic measure of psychological distress that includes 10 items addressing depression, anxiety, trauma, and physical problems. A score of ⩾25 indicates severe psychological distress.^19^

### Statistical analysis

Data were analysed using Microsoft Excel (version 2008, Microsoft Corporation, USA) and IBM SPSS Statistics (version 26, SPSS Inc, USA). Q-Q plots demonstrated normally distributed data for parametric statistical analysis to be performed. Where appropriate, analysis of variance (ANOVA) with post hoc testing was used to identify any differences between means of the OLBI, CORE-10 and COVID-19 experience sections of the questionnaire in different sociodemographic groups (e.g. education, geographical region, years of clinical experience and employment status). *T*-tests were used to further compare means, and the Pearson correlation coefficient was used to quantitively assess for any evidence of a linear relationship between variables. A value of *p* < 0.05 was used to determine statistical significance, and a value of *R*^2^ > 0.7 was used to determine strong linear correlation. Standard deviation is reported in the results as SD.

### Ethical considerations

This study received formal approval from City, University of London (reference: ETH2021-1240). Although all data were collected remotely and anonymously, participant well-being was considered with the provision of contact details for two UK-based mental health support groups where participants could self-refer and seek support. All participants confirmed their consent electronically via Qualtrics XM^TM^ before they were able to proceed to the questionnaire. All data were managed as per university guidance.

## Results

### Participant characteristics

In total, 138 sonographers actively participated in this study. Of those, 63.6% (*n* = 84) completed part 1 in full, 67.4% (*n* = 89) completed parts 2 and 3, and 66.7% (*n* = 88) completed part 4 of the questionnaire. Not all participants answered every question, which resulted in some missing data; however, all recorded responses were still included in the analysis. The average completeness for the entire questionnaire was 81%. Of those who answered the participant information questions (*n* = 89), the largest proportion of respondents identified as female (*n* = 86, 96.6%), of White/British/Welsh/Scottish/Northern Irish/Gypsy or Irish Traveller ethnicity (*n* = 77, 86.5%), between the ages of 51 and 60 years (*n* = 31, 34.8%) and working in the South East region of England (*n* = 20, 22.5%). Full participant characteristics are reported in Table 1.

**Table 1.** Participant characteristics.

Age group	21–30, *n* = 12 (13.48%) 31–40, *n* = 20 (22.47%) 41–50, *n* = 24 (26.97%) 51–60, *n* = 31 (34.84%) 61+, *n* = 2 (2.25%)
Gender	Female, *n* = 86 (96.63%) Male, *n* = 2 (2.25%) Prefer not to say, *n* = 1 (1.12%)
Ethnicity	White / British / Welsh / Scottish / Northern Irish / Gypsy or Irish Traveller, *n* = 77 (86.52%) Asian / Asian British, *n* = 4 (4.49%) Mixed / Multiple ethnic, *n* = 2 (2.25%) Other, *n* = 2 (2.25%) Black / African / Caribbean / Black British, *n* = 1 (1.12%) Prefer not to say, *n* = 3 (3.37%)
Education	University degree (postgraduate), *n* = 79 (87.00%) Diploma in Medical Ultrasound, *n* = 5 (5.00%) University degree (undergraduate), *n* = 3 (3.00%) Prefer not to say, *n* = 3 (3.00%)
Years of experience	0–5, *n* = 19 (21.35%) 6–10, *n* = 13 (14.61%) 11–15, *n* = 18 (20.22%) 16–20, *n* = 13 (14.61%) 21–25, *n* = 9 (10.11%) 26+, *n* = 17 (19.10%)
Professional memberships	Society of Radiographers, *n* = 79 British Medical Ultrasound Society, *n* = 40 Royal College of Midwives, *n* = 9 International Society of Ultrasound in Obstetrics and Gynecology, *n* = 2 Royal College of Nursing, *n* = 1 Other, *n* = 1 Prefer not to say, *n* = 1
Geographical location	England – South East, *n* = 20 (22.47%) England – North West, *n* = 13 (14.61%) England – South West, *n* = 13 (14.61%) England – East, *n* = 10 (11.24%) England – London, *n* = 9 (10.11%) England – East Midlands, *n* = 6 (6.74%) England – West Midlands, *n* = 5 (5.62%) England – Yorkshire and the Humber, *n* = 4 (4.49%) Wales, n = 3 (3.37%) Scotland, *n* = 2 (2.25%) Prefer not to say, *n* = 4 (4.49%)
Employment status	Full-time employment (NHS/public sector), *n* = 44 (49.44%) Part-time employment (NHS/public sector), *n* = 42 (47.19%) Part-time employment (private practice), *n* = 1 (1.12%) Other, *n* = 1 (1.12%) Prefer not to say, *n* = 1 (1.12%)

### Sonographers’ experiences of obstetric scanning during COVID-19

Of those answering the question (*n* = 107), most sonographers (97.2%, *n* = 104) reported using PPE (either employer provided or self-supplied) when scanning asymptomatic pregnant women or people. For symptomatic pregnant women or people, 97.6% (*n* = 83) of sonographers answering the question (*n* = 85) reported using PPE when scanning. There were 17 sonographers who reported they were not scanning symptomatic pregnant women or people at all. Sonographers’ opinions were sought on a range of issues using scales where 0 = negative response/impact or portrayal and 10 = positive response/impact or portrayal. First, sonographers were asked how safe they felt performing pregnancy scans during the pandemic, giving a mean score of 4.25 (SD = 2.58). When asked to rate the impact of COVID-19 on their scanning practice, the mean score was 6.40 (SD = 2.68). The impact of COVID-19 on communication with expectant parents was rated at an average score of 4.03 (SD = 1.87). Sonographers’ mean rating of the impact of COVID-19 on the overall parent experience of obstetric ultrasound was 3.27 (SD = 1.67).

### Portrayal of the sonographic profession in the news during COVID-19

**Figure 1.**
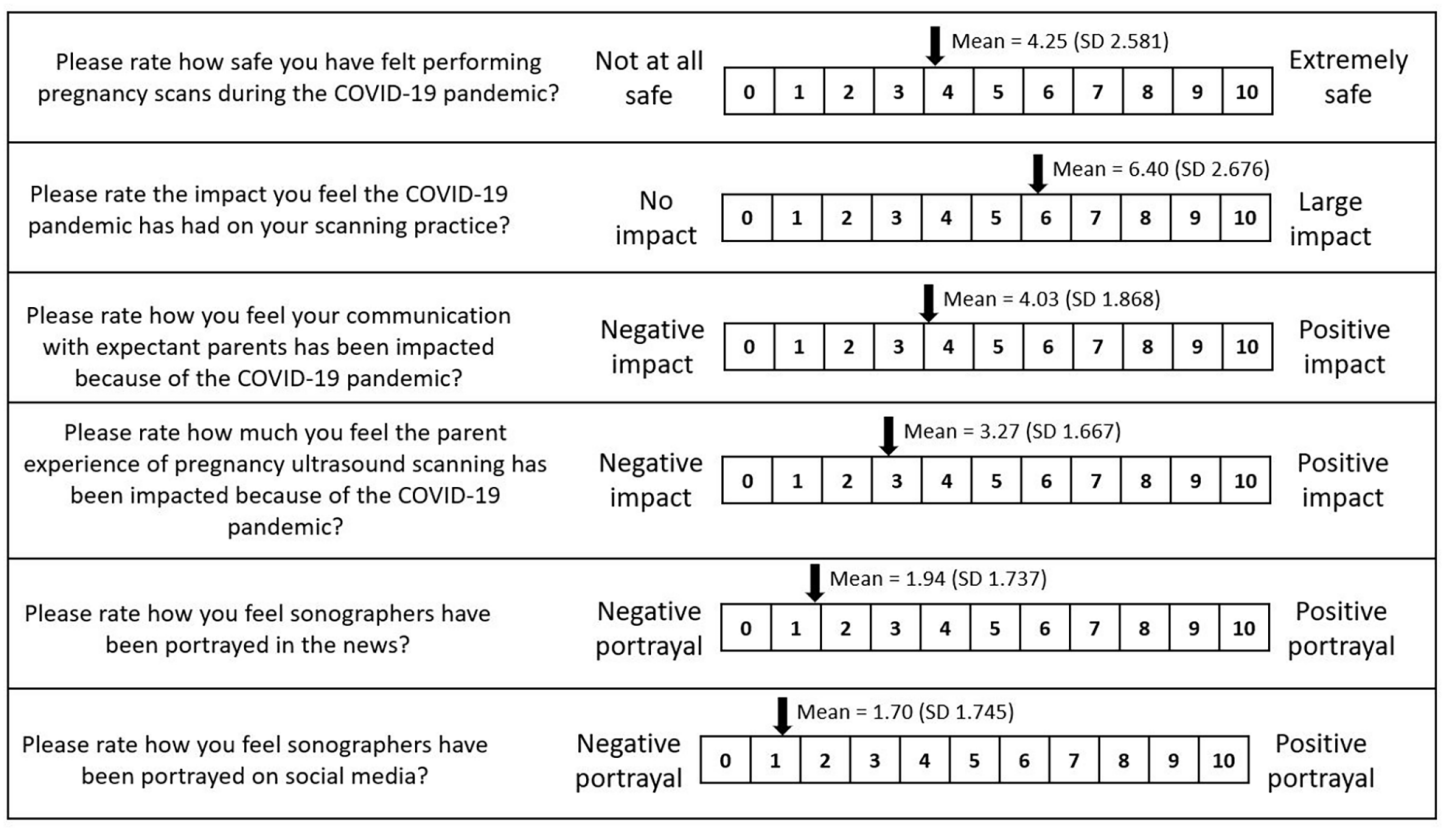
Impact of COVID-19 on sonographic practice.

**Figure 2.**
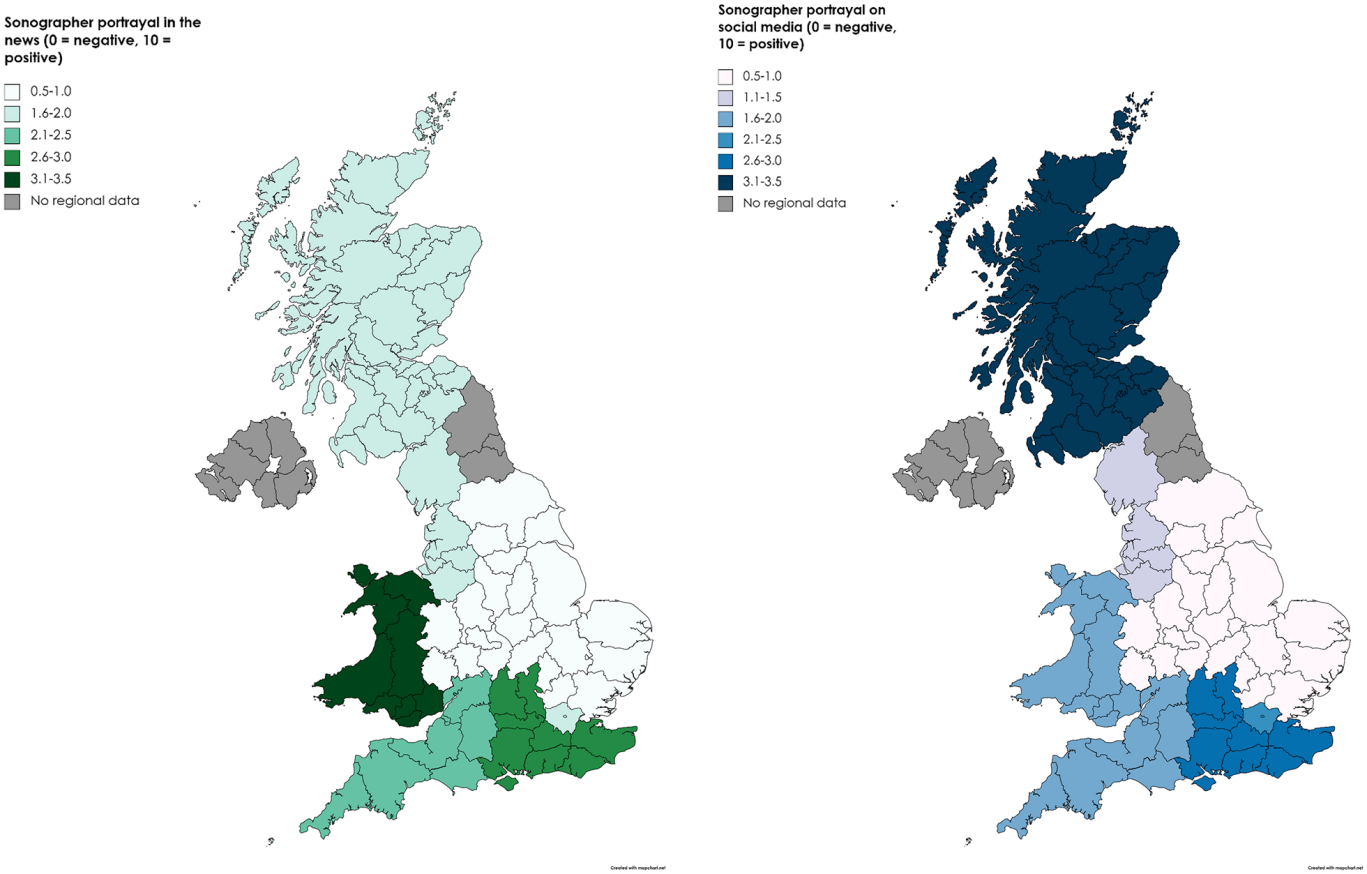
Geographical variation in perception of sonographer’s portrayal in the media.

When asked how they felt the profession had been portrayed in the news (e.g. newspapers and online press articles) during the pandemic, the sonographers’ mean score was 1.94 (SD = 1.74) (Figure 1). The lowest mean score was reported in the West Midlands (0.6, SD = 0.55) and the highest was in Wales (3.33, SD = 2.89) (Figure 2).

### Portrayal of the sonographic profession on social media during COVID-19

The mean score for the portrayal of the sonographic profession on social media (e.g. Twitter, Facebook) was 1.70 (SD = 1.75) (Figure 1). The lowest mean score was again reported in the West Midlands (0.40, SD = 0.55) and the highest in Scotland (3.50, SD = 2.121). For portrayal of the profession in both the news and on social media, the mean scores by geographical region did not exceed 3.5 (Figure 2).

A paired *t*-test showed that the mean difference in sonographer portrayal in the news and on social media was not significant (*p* = 0.110); however, a moderate positive correlation was noted between the scores (*R*^2^ = 0.427, *p* < 0.001). The perceived portrayal of sonographers in the news scored an average of 0.24 more positive than on social media (95% confidence interval (CI) (−0.055, 0.529)).

### Reliability analysis

Cronbach’s alpha showed good internal consistency of the OLBI for the eight items of the exhaustion dimension (α = 0.802) and acceptable internal consistency for the eight items of the disengagement dimension (α = 0.777). The reliability analysis performed on the 10 items of the CORE-10 showed good internal consistency (α = 0.881).

### Burnout (OLBI) and psychological distress (CORE-10)

Of a maximum 64 points, the mean total burnout (OLBI) score was 44.47 (SD = 7.60). The mean score for the exhaustion domain was 2.96 (SD = 0.49) and for the disengagement domain was 2.67 (SD = 0.48). The results showed 92.1% of sonographers (*n* = 82) met the burnout threshold for exhaustion (⩾2.25) and 91.0% (*n* = 81) met the burnout threshold for disengagement (⩾2.10). Geographical region, education, years of experience and employment status (e.g. full-time or part-time) did not appear to influence burnout scores in this study.

The mean CORE-10 score was 14.39/40 (SD = 7.99). This equates to mild psychological distress. No significant differences were identified between grouped participant characteristics and CORE-10 score.

**Figure 3.**
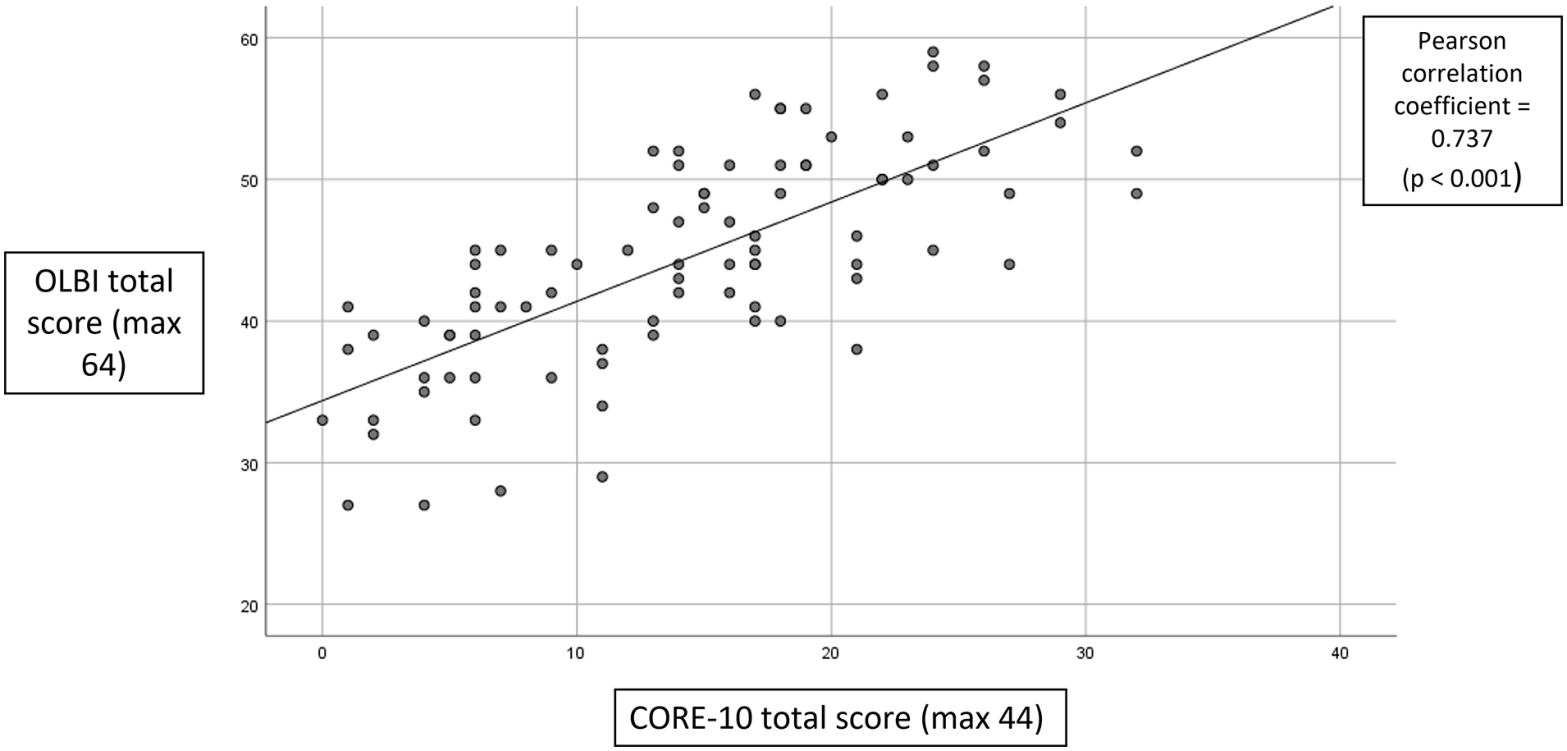
Correlation between OLBI and CORE-10 score.

The Pearson correlation coefficient demonstrated a statistically significant linear relationship between total burnout (OLBI) score and psychological distress (CORE-10) score (*R*^2^ = 0.543, *p* < 0.001). The magnitude of the association was moderate. This shows a positive trend between sonographers with a higher burnout score and higher levels of psychological distress (Figure 3).

### Sonographers’ experiences, burnout and psychological distress

The Pearson correlation coefficient was calculated to assess the association between different aspects of sonographer experience factors and the total burnout (OLBI) or distress (CORE-10) score. Statistically significant negative linear relationships were demonstrated between sonographers’ perception of safety and total burnout score (*R*^2^ = 0.198, *p* < 0.001) and distress score (*R*^2^ = 0.079, *p* = 0.008). A positive trend was observed between the sonographers’ perceived impact of COVID-19 on obstetric scanning practice and total burnout score (*R*^2^ = 0.044, *p* = 0.048). No other statistically significant associations were demonstrated.

### Impact of COVID-19 pandemic on sonographer satisfaction in role

Where 0 = not at all satisfied and 10 = very satisfied, the mean satisfaction in the sonographer role prior to the COVID-19 pandemic was 6.99 (SD = 2.01). Role satisfaction before the COVID-19 pandemic scored on average 2.87 points higher than during the pandemic (SD = 2.58, 95% CI (2.35, 3.39), resulting in a significant change in sonographer role satisfaction from before to during the pandemic (*t*_97_ = 10.988, *p* < 0.001). A significant, positive correlation between sonographers’ individual before and during pandemic role satisfaction scores was demonstrated (*R*^2^ = 0.145, *p* < 0.001). No differences were seen in role satisfaction between grouped participant characteristics using analysis of variance (ANOVA); however, statistically significant linear relationships were demonstrated between the change in satisfaction and total burnout (*R*^2^ = 0.157, *p* < 0.001), and psychological distress scores (*R*^2^ = 0.095, *p* = 0.003). In addition, statistically significant negative correlations were also demonstrated between respondents’ change in role satisfaction and sonographers’ portrayal in the media (*R*^2^ = 0.050, *p* = 0.028), portrayal on social media (*R*^2^ = 0.066, *p* = 0.011) and perception of safety (*R*^2^ = 0.148, *p* < 0.001).

### Impact of COVID-19 on working practice

**Figure 4.**
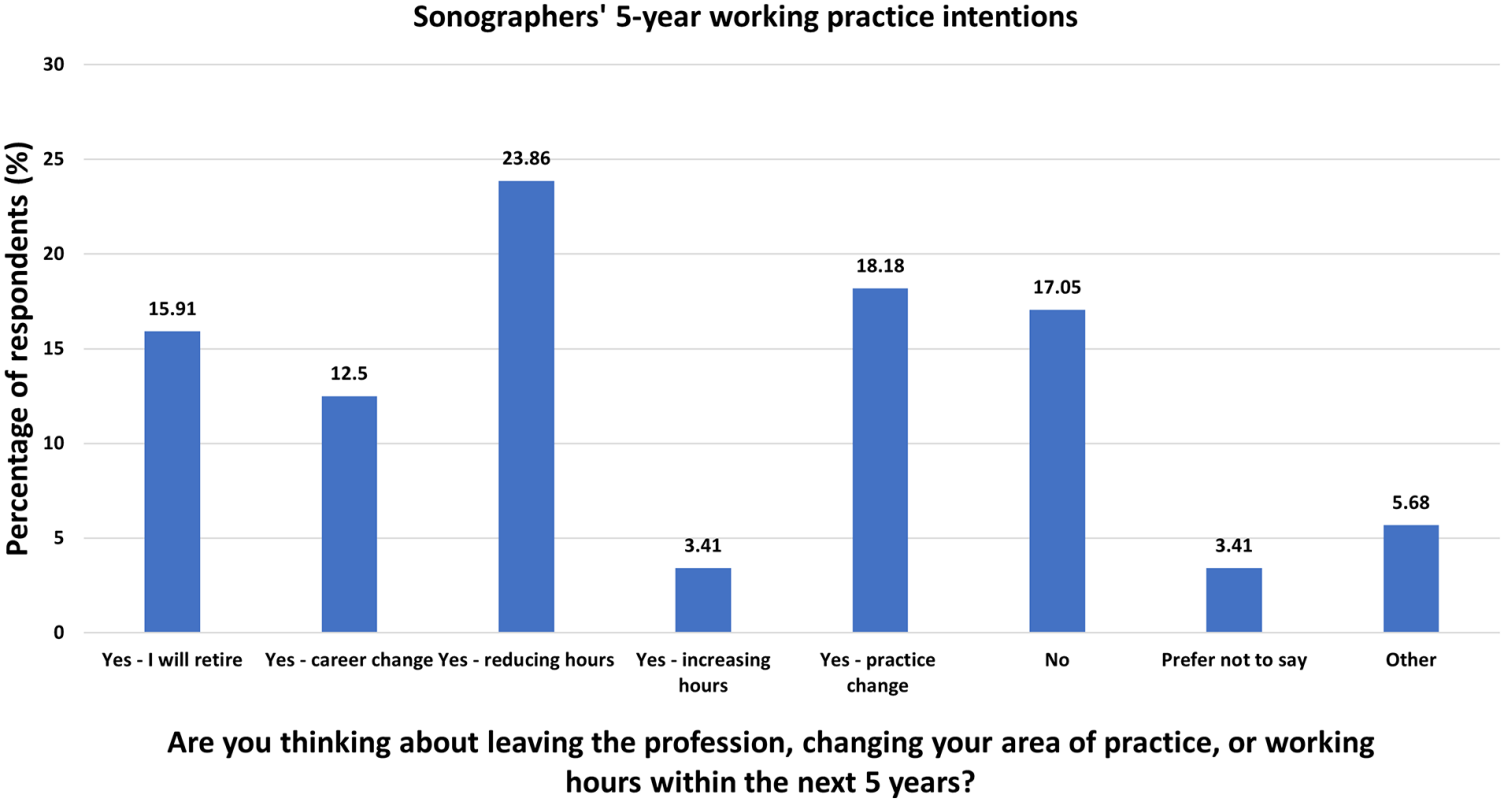
Sonographers’ 5-year working practice intentions.

Of the 88 sonographers who answered the question ‘Are you thinking about leaving the profession, changing your area of practice or working hours within the next 5 years?’, 73.9% (*n* = 65) responded ‘yes’ (Figure 4). Of these, 67.1% (*n* = 47) of sonographers said that their practice change would happen sooner than planned because of the COVID-19 pandemic. Nearly a quarter of sonographers (24.6%, *n* = 16) reported their intention to change their practice by no longer performing obstetric ultrasound examinations. Change of practice was weakly, positively correlated with psychological distress (CORE-10) score (*R*^2^ = 1.359E−4) and difference in role satisfaction before and during the pandemic (*R*^2^ = 0.012); however, neither were significant associations. Change of practice was weakly, negatively correlated with total burnout (OLBI) score (*R*^2^ = 0.010), although this was not significant either.

## Discussion

This study aimed to explore the impact of the COVID-19 pandemic on sonographers performing obstetric ultrasound examinations in the UK. Over 90% of sonographers in this sample who completed the OLBI met the burnout thresholds for exhaustion or disengagement. The findings of this study suggest a greater incidence of burnout among the sonographic workforce compared to similar studies using the OLBI to evaluate COVID-19-related burnout in healthcare professionals. For example, Denning et al.^20^ identified 67% of healthcare workers from across the UK, Poland and Singapore as being at high risk of burnout. Tan et al.^18^ reported 75.3% and 79.7% of healthcare workers meeting the threshold for exhaustion and disengagement, respectively. In both studies, healthcare workers were identified as doctors, nurses, allied health professionals and non-clinical/administrative staff. Higher burnout score was associated with being in a clinical role and redeployment to a new clinical area;^18,20^ however, the site of work (e.g. hospital, community, home-based) was not.^18^

Tan et al.^18^ also found that those who scored higher for burnout were also more likely to score higher for psychological distress, as demonstrated by a significant, positive correlation. This finding is supported by Chigwedere et al.’s^9^ systemic review, which observed a predictive relationship between high anxiety scores and burnout. In this study, a higher total burnout score was also significantly associated with a negative perception of the impact of COVID-19 on scanning practice. It was also demonstrated that sonographers who reported a large, negative change in role satisfaction before and during the pandemic were more likely to have higher total burnout and distress scores. This implies that reduced job satisfaction contributes to burnout and psychological well-being for sonographers. A similar relationship between job satisfaction and psychological distress in primary healthcare nurses was reported by Stefanovska-Petkovska et al.,^21^ who also noted a statistically significant association between negative job satisfaction and resignation. However, in this study, no significant association was demonstrated between change in role satisfaction post-pandemic and planned changes to practice. Statistically significant relationships (albeit weak) were observed between sonographers’ perceptions of feeling safe while scanning and total burnout and distress scores. A recent study reported elevated psychological distress in Israeli dentists and dental hygienists who were fearful of contracting COVID-19,^22^ which suggests this may have been an important moderator.^9^

### Impact of burnout on the sonographic workforce

In addition to the negative impact on individuals’ well-being,^6,9^ high levels of burnout within the workforce have several important implications for sonographic practice. An association between practitioners who score higher for occupational burnout and absenteeism has been reported.^16,23^ With the sonographic workforce vacancy rate at 12.6%^24^ and increased sickness rates from COVID-19 and through precautionary measures of self-isolation,^25^ additional absenteeism because of burnout is likely to further heighten the workload and subsequent job demands of other obstetric sonographers. This in turn may contribute to their increased exhaustion. Indirectly, burnout may also affect the sonographic team through its influence on working conditions, leading to dissatisfaction and disengagement with the work, and reduced organisational commitment.^6^ In this study, a significant decrease in sonographer role satisfaction (compared with perceived satisfaction pre-pandemic) was noted during the pandemic. One highly debated response to employee dissatisfaction is that of the Exit-Voice-Loyalty-Neglect (EVLN) typology, whereby in reacting to a problematic event, a worker may resign (exit), attempt to improve the situation (voice), wait to see whether the issue will be resolved (loyalty) or passively obstruct potential improvements, for example, through lack of interest (neglect).^26^ Of those answering the question in this study, over 70% of sonographers stated they were considering leaving or changing their practice within the next 5 years. As this was not found to be significantly associated with burnout, psychological distress or role satisfaction, this typology further highlights the negative impact of the pandemic on the workforce by the high proportion of sonographers with the intention to remove themselves from the clinical situation completely (exit) over other responses (e.g. voice or loyalty).

### Potential impact of burnout on provision of parent-centred care

Despite concerns regarding the physical barrier of PPE as a hindrance to effective patient–practitioner interaction,^27^ sonographers rated the impact of COVID-19 on their communication with parents as mildly negative to none in this study. Although this study was unable to directly assess the impact of burnout on parent care and outcomes, Freudenberger reported that regardless of the effort, burnout will affect how efficiently an individual can perform.^28^ High levels of burnout are associated with poor patient safety outcomes, including increased likelihood of errors,^29^ as well as low-quality patient interaction and care experiences.^10^ In this study, sonographers indicated that the pandemic had a moderate impact on their scanning practice and perceived a mildly negative impact on the parents’ experience of the ultrasound scan. The parents’ experience of obstetric ultrasound may be enhanced when they are actively involved in the scan;^30^ however, it is suggested that exhausted healthcare professionals may be more likely to view patient requests for interactivity as demanding.^31^ Repeated interactions that evoke feelings of cynicism over time can cause practitioners to withdraw and disengage in an attempt to conserve their emotional resources.^31^ This explanation is based on theories of social equity and reciprocity applied to healthcare settings, whereby a perceived imbalance in the patient–practitioner relationship (e.g. the caregiver feels their investment in the relationship is significantly greater than is reciprocated by the patient) actively contributes to burnout syndrome,^31^ further impacting care delivery.

### Reciprocity and role satisfaction

Reciprocation from patients through expression of gratitude has been shown to reduce burnout in nurses.^32^ In this study, a negative correlation was demonstrated between how respondents felt the sonographic profession had been portrayed in the news and on social media during the pandemic, and their change in role satisfaction during the pandemic. Interestingly, significant differences were also found between geographical regions and the sonographer’s media portrayal, suggesting there may have been areas in the UK where the media attention was more concentrated. During the pandemic, other healthcare professionals also received a lot of public attention; however, much of it was in praise of ‘heroic’ frontline workers (e.g. #clapforheroes). This narrative has been questioned for its potential adverse psychological effects on staff, causing stress through increased moral responsibility,^33^ as well as implying that reciprocal social obligations are unrequired.^34^ In addition, persistent unrealistic expectations about the interpersonal relationship between staff and their patients can also cause imbalance of reciprocity when they are not met, leading to burnout of the individual. This can affect the whole team via the socially induced model of burnout transmission.^35^ Sonographic teams are typically small and work closely together; thus, there is a greater chance of being directly exposed to and mirroring a colleague’s symptoms of exhaustion or disengagement, or reaching burnout because of a change in work conditions initiated by a colleague with burnout.^35^ Many burnout interventions suggested in the published literature focus on promoting individual well-being and resiliency, with limited evidence of efficacy demonstrated.^33^ Therefore, interventions that address occupation-specific factors contributing to burnout may be more successful in easing exhaustion and disengagement. As per Demerouti et al.^2^ these should aim to reduce job demands (e.g. improving the physical working environment or varying tasks to balance the physical workload) while providing greater job resources (e.g. personal support from supervisors, opportunities for career development). This model suggests that significant action at organisational level may now be required to alleviate pandemic-induced burnout.

### Strengths and limitations of study

A strength of this study was the use of the validated OLBI and CORE-10 tools. These demonstrated good reliability within the study and have clearly defined thresholds which were used to aid interpretation of results. The sample size may be considered relatively small and not representative of the UK obstetric sonographer population; however, it was comparable with other UK sonographer studies.^3,36^ While the results focus on, as needed, the peak of the COVID-19 pandemic, the cross-sectional design of this study limits conclusions of causality.^37^ The self-selected and self-reported participation may skew the results towards those motivated to share negative personal experiences. In addition, the results are susceptible to common method bias (a known limitation of questionnaire design where the same tools are used to collect all data), which can result in artefactual estimates of the relationships between constructs.^38^ While the homogeneity of participant characteristics for gender and ethnic identity improves confidence that the findings accurately represent the study sample, the results cannot be generalised to a wider, more heterogeneous population and are therefore limited beyond this specific demographic.^39^

### Future research

A follow-up survey to compare sonographer well-being and role satisfaction after the pandemic would be beneficial to determine whether self-reported burnout scores reduce when the additional stressors of COVID-19 are diminished. This may also help to identify any limitations in the study data incurred through over-reporting of negative personal experiences during the pandemic. Considering an alternative method of data collection, for example, using impartial assessors to determine burnout, may also be more accurate than using self-reported scores. Qualitative analysis of free-text responses collected as part of this questionnaire may provide deeper insight into sonographers’ experiences of performing obstetric ultrasound scans during the COVID-19 pandemic to help further inform the quantitative observations. Further research could also consider the impact of individuals’ differences (e.g. personality traits, home demands) on burnout and psychological well-being.^6^

## Conclusion

Most respondents in this study met burnout thresholds for exhaustion and disengagement. Sonographers with a higher burnout score also demonstrated higher levels of psychological distress and negative changes in role satisfaction, which has implications for the delivery of parent-centred care. Sonographers perceived the pandemic to have had a moderate impact on their immediate scanning practice; however, the findings of this study suggest that the long-term impact on the workforce is yet to be fully realised as demonstrated in the high proportion of respondents considering a change in their clinical practice within the next 5 years. Urgent interventions are therefore required to mitigate the consequences of burnout within the profession, such as those to reduce job demands and increase resources, improve sonographer role satisfaction, and enhance and promote positive relationships between sonographers and expectant parents in the scan room.
